# Multiple Lattice Model for Influenza Spreading

**DOI:** 10.1371/journal.pone.0141065

**Published:** 2015-10-29

**Authors:** Antonella Liccardo, Annalisa Fierro

**Affiliations:** 1 Physics Department, Università degli Studi di Napoli “Federico II”, Napoli, Italy; 2 Istituto Nazionale Fisica Nucleare (INFN) - Sezione di Napoli, Napoli, Italy; 3 Consiglio Nazionale delle Ricerche (CNR) - Institute Superconductors, oxides and other innovative materials and devices (SPIN), Napoli, Italy; Shanxi University, CHINA

## Abstract

Behavioral differences among age classes, together with the natural tendency of individuals to prefer contacts with individuals of similar age, naturally point to the existence of a community structure in the population network, in which each community can be identified with a different age class. Data on age-dependent contact patterns also reveal how relevant is the role of the population age structure in shaping the spreading of an infectious disease. In the present paper we propose a simple model for epidemic spreading, in which a contact network with an intrinsic community structure is coupled with a simple stochastic SIR model for the epidemic spreading. The population is divided in 4 different age-communities and hosted on a multiple lattice, each community occupying a specific age-lattice. Individuals are allowed to move freely to nearest neighbor empty sites on the age-lattice. Different communities are connected with each other by means of inter-lattices edges, with a different number of external links connecting different age class populations. The parameters of the contact network model are fixed by requiring the simulated data to fully reproduce the contact patterns matrices of the Polymod survey. The paper shows that adopting a topology which better implements the age-class community structure of the population, one gets a better agreement between experimental contact patterns and simulated data, and this also improves the accordance between simulated and experimental data on the epidemic spreading.

## Introduction

Contact patterns among individuals are crucial in studying the spreading of close-contacts infectious diseases. This idea has been inspiring for an extensive literature [1–7] and it is at the base of the *social contact hypothesis*, which explicitly assumes that the number of potentially infectious contacts of an individual is proportional to the number of self reported contacts [8, 9]. Moreover, at individual level, *age* is a fundamental aspect with respects to the risk of infection and, consequently, the age structure of a population strongly influences the disease spreading. Individuals of different age-classes have, indeed, different hygienic habits and frequently different susceptibilities to the infection due to partial pre-existing immunity, as it happened with the H1N1 pandemic [10–12]. Data on age-dependent contact patterns also reveal that individuals of different age have very different social behaviors with relevant implications for the spreading of infectious disease in different age classes [13]. The school environment, for instance, typically favors the epidemic spreading among young people more than the work environment does for adults [14–17]. This justifies the increasing interest in collecting data on contact patterns [18–20] also adopting new technologies, as for instance those based on the use of sensors to detect proximity among people [21–23]. Thus, a model aimed at reproducing the epidemic spreading should be able to properly take in account the specificity of each age-class both from the biological and social point of view.

Diary-based large scale surveys also highlight the homophily of the social network: people strongly prefer contacts with similar people and specially with individuals belonging to the same age-class, i.e they have an assortative behavior, as shown by the large diagonal elements of the contact patterns matrices collected by the Polymod survey all over Europe [9].

Behavioral differences among age classes and commonalities within age-classes, together with the human tendency to assortative behaviors, naturally point to the existence of a community structure in the population network, in which each community can be identified with a different age class. The community structure is a peculiar feature of social networks with respect to the non social ones, and it has been shown to be responsible both for the typical clustering [24] and the positive correlations (i.e. assortative mixing) of the social networks [25], and viceversa [26].

The focus on the age-distributed contact patterns of individuals, as the leading key to interpret and reproduce the contagion process, has been the *leitmotiv* of some recently published papers [27–29], based on the assumption that a detailed microscopic knowledge of the population structure, although powerful and fascinating, is not strictly necessary in order to reproduce the spreading of an infectious disease, which is instead essentially regulated by the average contact patterns of individuals. Under this hypothesis, improving the agreement between simulated and experimental data on contact patterns, one obtains a better agreement also on the epidemiological previsions. In particular, in those papers, the population was entirely distributed on a D-dimensional lattice, representing the dynamic contact network of individuals, and the dynamics on the lattice was governed by an attractive interaction between individuals belonging to the same age-class, aimed at reproducing the assortative behavior of the social network. In [28, 29] the parameters that regulate the pattern dynamics were fixed by fitting data on the age-dependent daily contact numbers, furnished by the Polymod survey. A simple stochastic SIR transmission model, with a nearest neighbor interaction, age-specific susceptibilities and some very basic adaptive mobility restrictions, completed the model. The impact of self-initiated behavioral changes that reduce the susceptibility to the disease, altering the transmission parameters, was also introduced in Ref. [29]. The model, validated against the age-distributed Italian epidemiological data for the influenza A(H1N1) during the 2009/2010 season, reproduces the epidemiological data during the epidemic peak for all the age-classes. However, deviations were found in the descendant phase, mainly for young people.

One of the limits of this lattice-gas model is that, even if it is based on an attractive interaction among individuals of the same age class and adopts age-specific susceptibilities, the age-ingredient is not fully exploited in it. Indeed, as already outlined in Ref. [28] by the authors, the model parameters were fixed by the minimal requirement that the simulated data should reproduce the overall numbers of daily contacts in each age-class, without discriminating among the age of the encountered people: i.e. no explicit constraints on the contact matrix, which gives the full information about the mixing between different age classes, were imposed. This limit is related to the model structure. Indeed, as previously said, diary-based large scale surveys indicate that young people have a much more intense social activity than elderly people and children, but at the same time they have a strong tendency to assortativity. This circumstance could not be reproduced within the previously mentioned model because the mobility and the assortativity were regulated by the same parameter (*β*_*age*_), falling in two opposite regimes (*β*_*age*_ → 0 corresponding to the high mobility regime and *β*_*age*_ → ∞ corresponding to the high assortativity regime). One needs to rethink the dynamic on the network, by introducing some further parameters in order to keep together both the constraints on assortativity and mobility.

**Fig 1:**
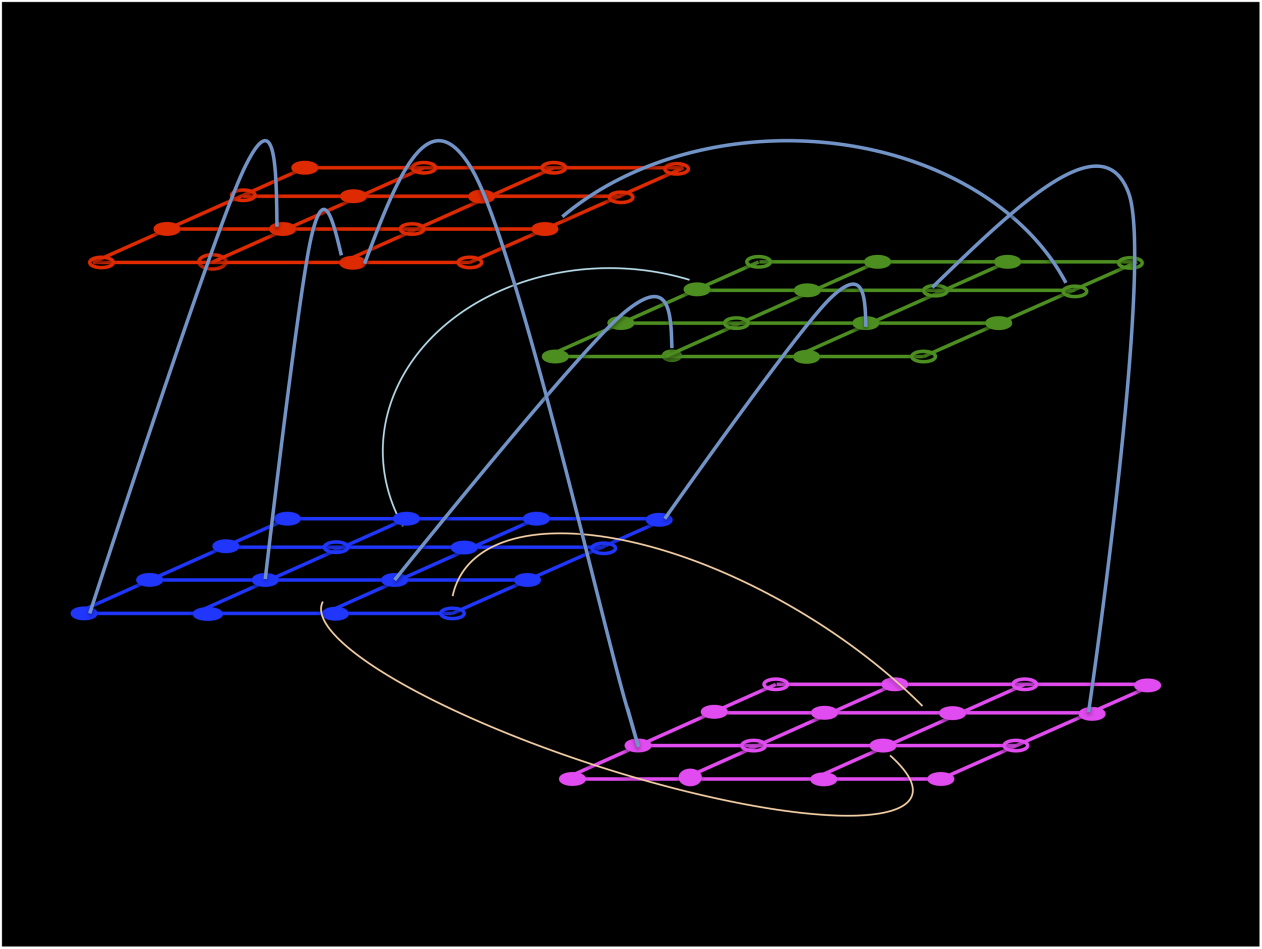
Multiple lattice structure—2D representation.

In order to overcome this limit, in the present paper, we introduce the concept of modularity as a structural element of the contact network. The interest in the study of the epidemic spreading on complex networks with a community structure has increased in the last years [30–32]. In the present paper we construct a multiple lattice model in which the population network is a priori divided in 4 different communities, one for each age class (see Fig 1). Each community occupies a specific age-lattice, with a certain percentage of occupation and individuals are allowed to move freely to nearest neighbor empty sites on the age-lattice. Different communities are connected with each other by means of inter-lattices edges, with a different number of external links connecting different age-lattices. Two individuals of the same age class are connected if they occupy two nearest neighbors sites. Two individuals of different age classes are connected if there is an edge among the sites in which they are positioned. This structure allows to fix separately the contacts among different age classes and those internal to a specific age class, each of them being regulated by a different parameter. In so doing one can fit the whole contact patterns matrix, exploiting completely the age-dependent information encoded in the Polymod data.

## Materials and Methods

### Validating Data

The evaluation of the modularity parameter [33] on simulated contact data highlights the existence of a strong community structure inside the network, giving a clear indication about the modularity structure of the Italian population network, implicitly encoded in the Polymod data.

On such model of the social interaction, we turn on the epidemic spreading, with a stochastic simple SIR model, with very basic adaptive rules, and compare the predictions with the H1N1 pandemic data. In so doing, we test the robustness of the social contact hypothesis approach by exploring further topologies for the contact network. Interestingly, we find that the setup that better allows to reproduce the contact patterns, such as the multiple-lattice one, gives better results also for the epidemic spreading, if compared with the single lattice setup.

In conclusion, the paper aims to show that adopting a topology, which better implements the age-class community structure of the population, one gets a better agreement between experimental contact patterns and simulated data and this also improves the accordance between simulated and experimental data on the epidemic spreading.

Data on social contacts are those collected by diary-based large scale survey of epidemiologically relevant contact patterns. As emphasized in [5], the contact diary methodology [8, 9, 34], in spite of its limitations due to biases [35, 36] in recording the contacts from the participants, or in reporting the duration or the type of contact (conversation, touch, skin to skin, kisses), still remains a fundamental one in the epidemiological analysis. Among the contact diary studies, the Polymod one is accounted to be the largest and more relevant study of this kind performed in 8 EU countries. Thus we keep it as the reference study for the contact patterns data also in this paper. The country dependent contact matrices, *m*_*ij*_ (i.e. the average number of daily contacts that an individual in the age class *j* has with individuals in the age class *i*) were obtained from the self reported number of social contacts in each age class. The dominant feature of these contact matrices, all over Europe, is the large diagonal elements (larger in the 5–24 age class than in the adult ones), which indicate that individuals prefer contacts with individuals belonging to the same age class, i.e they have an assortative behavior.

The epidemiological data adopted to validate the model are the H1N1 pandemic data during the season 2009/2010 in Italy,. This choice facilitates the comparison with previous models that were validated against those data. Furthermore, the peculiarity of the H1N1 pandemic, was that it occurred in a different period from the seasonal influence, and this circumstance reduced the overlap effects of different viruses in the data collection.

Epidemiological data in Italy are furnished by Influnet, the surveillance system, coordinated by the Ministry of Health in collaboration with the Istituto Superiore di Sanità (ISS), the Interuniversity Centre for Research on Influenza (CIRI), the Regional Health Departments, the general practitioners and pediatricians and some University Laboratories, with a coverage of at least 1–5% of the population. Data are reported in terms of aggregated weekly number of patients seen with influenza-like illness (ILI), and are collected through a sentinel networks of physicians and pediatricians. Age-specific incidence data of H1N1 pandemic during the season 2009/2010 are available in the report [37] with a partition in four age classes (0–4, 5–14, 15–64, over 65 years old). Apart fluctuations, the number of physicians participating to the surveillance during the H1N1 epidemic, and correspondingly the number of patients, were stable during the period between 40-th and 53-th week, considered for our simulations.

### Multiple Lattice Structure and Contact Network Dynamics

The age-distributed population is located on 4 different lattices, one for each age-class, as shown in Fig 1. In particular, each lattice *a* is occupied by *N*_*a*_ individuals with *a* = 1, 2, 3, 4, corresponding respectively to the age groups, 0–4, 5–14, 15–64 and over 65 years old. The lattices represent the dynamic contact network of individuals and not the physical space, i.e. no notion of distance is defined on it. The lattices are not fully occupied, each lattice being characterized by an occupation percentage equal to *ρ* × *ρ*_*a*_, where *ρ*_*a*_ are the age class densities and *ρ* is an arbitrary constant of proportionality between 0 and 1. The choice of *ρ* only affects the speed of the simulations. In the following, we fix *ρ* = 0.5 for all the lattices. Different lattices are connected with each other by external links that allow for interactions among different age-classes, as depicted in Fig 1. The external links that connect sites on different lattices, are kept fixed during the dynamics. The existence of empty sites allows individuals to move on the lattice and, in so doing, to change the identities (and eventually the number) of their simultaneous contacts both internal, i.e. with individuals of the same age class, and external (with individuals belonging to different age classes). By moving at random from a site to a nearest neighbor empty site, an individual moves from a certain environment/social group to another (e.g. from work to home).

The focus of the present paper is to check to what extent the predictivity of the epidemiological model can be improved by including a community structure into the network model and, in particular, by adopting the four lattices setup, which indeed allows to improve the adherence of the simulated age-dependent contact patterns with the one registered by the large scale surveys on social contacts. Our aim is to reproduce (as better as possible) all the entries of the contact matrix *m*_*ij*_ and not only the quantity $C_{j}=\sum_{i=1}^{4}m_{ij}$, which instead represents the total number of contacts that an individual of age-class *j* has on average in one day with other people, independently of the age-class to which they belong. One can also define the matrix *p*_*ij*_, whose element gives the fraction of the contacts of the age-class *j* with individuals belonging to the age-class *i*, i.e. *p*_*ij*_ = *m*_*ij*_/*C*_*j*_. The information about the assortativity is encoded in the diagonal elements of this matrix: the higher is the value of *p*_*ii*_, the higher is the assortativity of the age-class *i*.

In order to reproduce the assortativity, in the previous models [27–29], the interactions were governed by an attractive potential among individuals of the same age class, encoded in the following transition probability

$$T\left(1\to 2\right)=e^{-\beta_{age}\left|\Delta N_{age}\right|}$$ (1)

with Δ*N*_age_ being the increment of the number of nearest neighbors of the same age class in moving from site 1 to site 2, and the temperature parameters *β*_age_ represent the age-dependent inverse mobilities, and play the role of assortativity regulators. Those parameters were fixed by requiring the overall daily number of contacts to reproduce the corresponding Polymod data for the *C*_*j*_ in each age class. The total daily contact numbers, *C*_*j*_, obtained with the single lattice model were in good agreement with the Polymod data, but the assortativity of Polymod and the *m*_*ij*_ matrix elements were in general not properly reproduced. In the discussion section of [28] authors argued that this is a limit to be related to the double role of the parameter *β*_age_: on one hand, it works as a regulator of the assortativity and, on the other hand, it regulates the overall number of contacts. In particular, the high assortativity regime is obtained with *β*_age_ → ∞ but, in this limit, the mobility of individuals strongly reduces. In particular, the 5–14 age-class has the peculiarity to be at the same time, the most assortative and the most dynamic age-class. For this reason, it is hard to reproduce these two features within the same parameter. For further details on the role of *β*_age_ in the dynamics of individuals, we refer to Ref. [28].

In order to overcome this limit, in the present model, we follow a different approach. In particular, individuals move at random on each age class, according to an age class mobility (*mob*_*a*_) which is fixed by requiring the internal number of contacts (*m*_*ii*_) to reproduce the corresponding Polymod data, for each of the 4 age-classes considered. Furthermore, the number of external links that connect different lattices are also calibrated on the matrix elements *m*_*ij*_ of the Polymod survey. In particular, we extract them from a Gaussian distribution with symmetric parameters (*μ*_*ab*_, *σ*_*ab*_), with *a*, *b* running on the age groups. These parameters are fixed in order to reproduce the off diagonal contact matrix terms.

Notice that the Polymod contact pattern matrix is not symmetric by itself, because the population answering to the survey is not closed (people that answer the survey meet also people that are not involved into the survey). However, the Polymod matrix can be adjusted for reciprocity. The simulated data, instead, refer to a closed population and thus they produce symmetric contact patterns, *c*_*ij*_. In order to compare with the Polymod matrix, one has to divide the number of contacts *c*_*ij*_ by the number of individuals in each class (*N*_*i*_ and *N*_*j*_), obtaining the two distinct entries of the simulated matrix *m*_*ij*_ = *c*_*ij*_/*N*_*i*_ and *m*_*ji*_ = *c*_*ij*_/*N*_*j*_.

For what concerns the external links, they are distributed at random and satisfy the constraint *N*_*ab*_ = *N*_*ba*_, with *N*_*ab*_ being the overall number of external links between lattice *a* and lattice *b*.

### Baseline Transmission Model

The baseline transmission model is a SIR stochastic model, in which each individual can be healthy without immunity (i.e. susceptible, S), infective (I) or healthy with immunity (i.e. recovered, R). An internal degree of freedom for the healthy/infective status (*I*_*i*_ = 0, 1) is associated to each individual, and a further degree of freedom for susceptible/immune status (anti_*i*_ = 0, 1) is attributed to healthy individuals. After a potentially contagious contact with an infected nearest neighbor, a susceptible (*I*_*i*_ = 0, anti_*i*_ = 0) becomes infected (*I*_*i*_ = 1, anti_*i*_ = 0) according to her/his specific age class susceptibility, *S*_*a*_.

The infective individual goes through an asymptomatic phase, followed by a symptomatic one. During the epidemic, some disease adaptive rules are over-imposed:

- Infected individuals typically stay at home during the manifestation of symptoms, reducing their contact network essentially to the family. This tendency is implemented in the model by imposing that, at time *T*_*s*_ (where *s* stays for stop or symptoms) after the contraction of the infection, the infected individual stops and does not move until she/he recovers.
- Susceptible individuals tend to avoid contacts with the infected ones during their symptomatic phase. This tendency is implemented by imposing that on each lattice the empty sites which are nearest neighbor to symptomatic infected individuals or connected by inter-edges to infected individuals on different age-lattices, are interdict. In other words, symptomatic infected individuals can only infect their susceptible nearest neighbors at the stop time, *T*_*s*_ (i.e. the family in our simplified model).
- The neighbors of infected individuals can move without any restriction.

After a time *T*_inf_ since infection, the infected individual acquires permanent immunity (i.e. develops antibodies), changing the internal degree of freedom (*I*_*i*_, *anti*_*i*_) from (1, 0) to (0, 1), and starting to move again. Each infected individual has her/his own infective period. The infectivity is taken to be constant during the disease.

This simplification corresponds to disregard the effect of the viral load in the infection process, which is indeed entirely ascribed to the immunological status of the susceptible individual. To disregard the viral load in the transmission process is acceptable for highly infective disease, as the influenza pandemic. In any case, the model can be easily upgraded in order to overcome this simplification, introducing a variable infectivity and a susceptible-infector dependent transmission rate.

### Numerical Simulations

We consider 4 different numerical experiments (listed in Table 1) on lattices with dimension *D* = 3, 4, 6, 8, respectively. The linear size of the lattice, *L*(*D*), are fixed in order to have roughly the same population size for each dimension (∼ 1.500.000 individuals). For each experiment, we simulate *n* = 30 independent processes, initialized with different random seeds, and the quantities of interest are obtained averaging over these independent realizations of the system. The dimension *D* fixes the maximum number of simultaneous contacts that an individual can have within her/his age community. Here, we choose to work with a Neumann neighborhood (i.e. with 2*D* nearest neighbors). Periodic conditions are fixed on the lattice boundary.

**Table 1:** List of Numerical Experiments.

**Simulations**	**D**	**L**	**week/MCS**
**Experiment 1**	3	125	420
**Experiment 2**	4	38	315
**Experiment 3**	6	11	196
**Experiment 4**	8	6	140

Values of the parameters in the 4 different numerical experiments realized. The linear size of the lattice, *L*, is fixed in order to have roughly the same number of individuals (∼ 1.500.000) for each dimension, *D*. The conversion factor, necessary to reproduce the dynamics, depends on the lattice topology.

For the sake of clarity, we summarize here the main steps of the numerical simulations:

1. *Construction of the multiple lattice structure*—We consider four lattices of dimension *D* and connect each pair of lattices by external links in the following way. A site *j* in the lattice *a* is connected with a number *x*_*ab*_(*j*) of randomly chosen sites in the lattice *b*, which follows the Gaussian distribution $\exp(-{\left(x_{ab}\left(j\right)-\mu_{ab}\right)}^{2}/2\sigma_{ab}^{2})$.
2. *Thermalization of the system*—Each lattice *a* is occupied by *N*_*a*_ individuals. As reference population, we consider the Italian one, whose age-class distribution is given in Table 2. At each step, for each lattice *a*, a particle is randomly chosen and moved with probability *mob*_*a*_ to a randomly chosen nearest neighbor empty site in the lattice *a*. The system quickly reaches equilibrium. In this state the contact matrix *m*_*ij*_ is evaluated. The parameters *mob*_*a*_ are fixed in order to reproduce the diagonal contact matrix terms *m*_*ii*_ of the Polymod survey and the parameters (*μ*_*ab*_, *σ*_*ab*_) to reproduce the off diagonal contact matrix terms *m*_*ij*_. The mobility *mob*_*a*_ can be chosen independently on the lattice dimension *D*, the parameters (*μ*_*ab*_, *σ*_*ab*_) being instead dependent on *D*.
3. *Influenza spreading*—Once the multiple lattice structure is constructed and the mobilities fixed, the SIR stochastic model is applied to simulate the influenza spreading. Again, at each step, for each lattice *a*, a particle is randomly chosen and moved with probability *mob*_*a*_ to a randomly chosen nearest neighbor empty site in the lattice *a*. The state of the particle, given by the parameters (*I*_*i*_, *anti*_*i*_), is controlled. An infective particle (i.e. *I*_*i*_ = 1, *anti*_*i*_ = 0) may infect susceptible neighbor particles (i.e. *I*_*j*_ = 0 and *anti*_*j*_ = 0) in the lattice *b* (with *b* either different or equal to *a*) with probability *S*_*b*_, i.e. *I*_*j*_ becomes 1 with probability *S*_*b*_. Two particles in different lattices are considered neighbors if they are connected by an external link. At time *T*_*s*_ after the contraction of the infection, infective particle stops and does not move until time *T*_*inf*_ since infection is reached. The neighbor sites of a particle in this state (i.e. symptomatic infected) are interdicted. After a time *T*_*inf*_ since infection, the internal degree of freedom *I*_*j*_ becomes 0 and *anti*_*j*_ becomes 1.

In our simulations, the time unit is the Monte Carlo Step (MCS), that corresponds to the time interval necessary to ensure that each particle attempts to move on average one time. In order to reproduce the Polymod data, we also have to fix the relation between the MCS and the Polymod time unit (i.e. 1 day). The collapse of the numerical data onto the experimental ones is obtained by suitably fixing the time conversion factor. In particular, the number of MCS corresponding to 1 day reduces with increasing the lattice dimension (see Table 1). This is consistent with the fact that higher dimensional topologies imply a potentially higher number of simultaneous nearest neighbors. As a consequence, in higher dimensions the particle can reach the appropriate number of new contacts, expected in one day, with less movements. Let us notice that the role of the parameter D was stronger in the single lattice set-up ([28]), where indeed the parameter D significantly influenced the efficiency of the attractive interaction among particles of the same age-class, and thus the contact patterns. In the present set-up, instead, particles interact only via the excluded volume. The dynamics within each age-class lattice is simply governed by the mobility parameters that are fixed once for all in each dimension.

**Table 2:** Age-Class Parameters.

**age class (*age*)**	*ρ*_**a**_
**0–4**	0.048
**5–14**	0.093
**15–64**	0.659
**+65**	0.20

Distribution of the Italian population in age classes. ISTAT—2009

Notice that, although the multiple lattice structure is static, since the external links between the sites of different lattices are fixed once for all, the contact network is dynamics, since a particle may move from one site to another one in the same lattice, changing its neighbors.

## Results

### Contact Patterns

**Fig 2:**
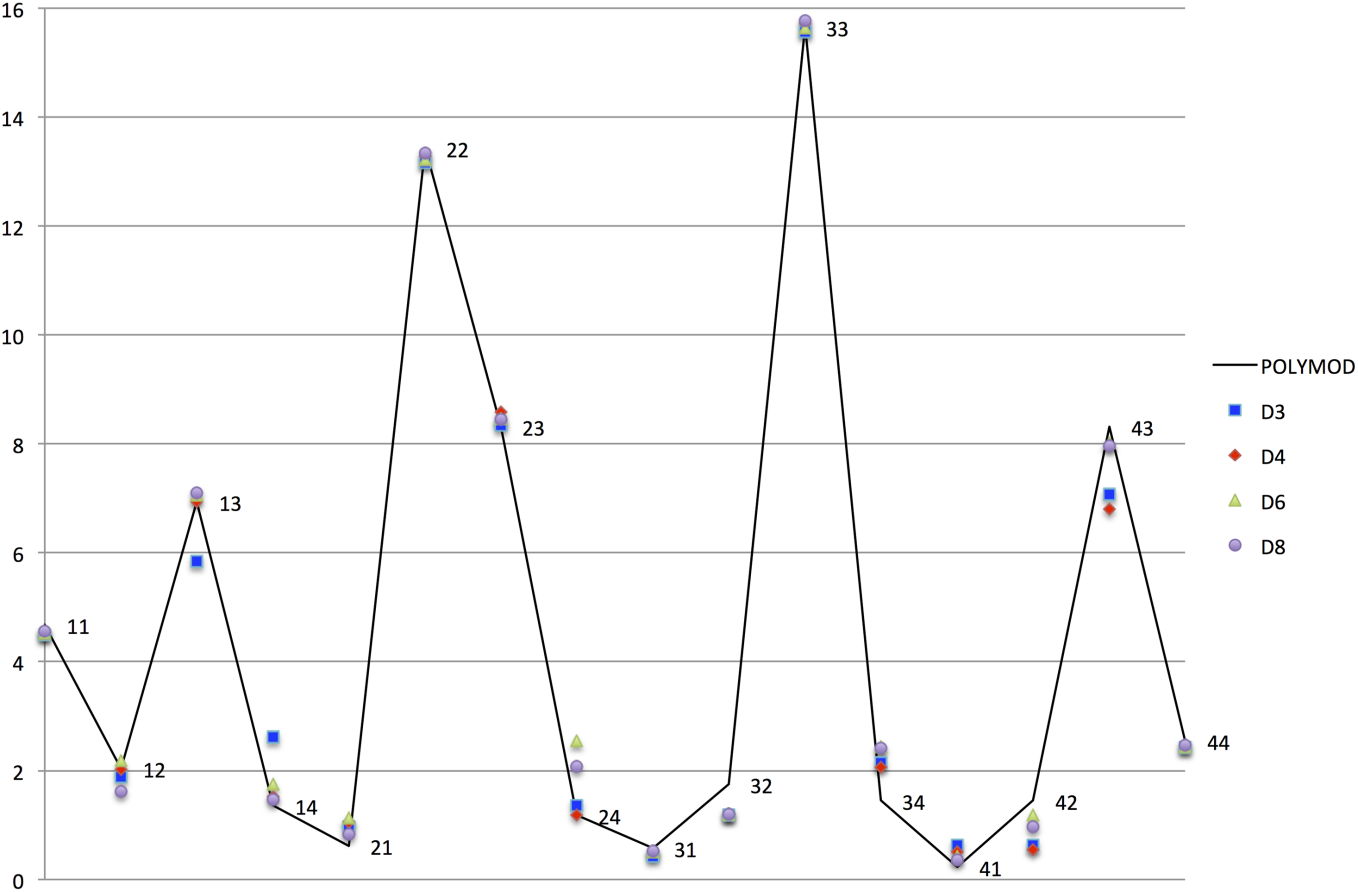
Contact patterns. The labels *ab*, with *a*, *b* ∈ {1, 2, 3, 4}, refers to contacts between age class *a* and age class *b*.

In Fig 2, we compare the Polymod data and the simulated values of the contact matrix, *m*_*ij*_, for each value of *D*. The *χ*-square test performed on the contact matrix are reported in Table 3. We see that the tests are quite good in all the experiments, and they give better results if compared with those obtained in [28] where only the daily overall contacts were fitted.

**Table 3:** *χ*-square test.

**Simulations**	**D**	**Contact Data**	**Epidemic data (9 points fit)**	**Epidemic data (11 points fit)**
**1 lattice**	6	0.1500	0.683	0.114
**Experiment 1**	3	0.9995	0.988	0.529
**Experiment 2**	4	0.9998	0.996	0.913
**Experiment 3**	6	0.9999	0.999	0.956
**Experiment 4**	8	0.9999	0.999	0.966

Values of the *χ*- tests for the contact data and epidemic data in the 4 numerical experiments realized, compared with those obtained in Ref. [28] by fitting the total contacts.

In order to explore the community structure of the simulated network we evaluate the modularity of the resulting network [33], defined as

$$Q=\frac{1}{2m}\;\sum_{ij}\;(A_{ij}-\frac{k_{i}k_{j}}{2m})\;*\;\delta \left(c_{i},c_{j}\right)$$ (2)

where ∑_*ij*_ runs over all the sites occupied, *m* is the total number of edges in the network, *A*_*ij*_ is the adjacency matrix (which takes value 1 if there is an edge between site *i* and site *j*, otherwise is 0), *k*_*i*_ is the vertex degree and *δ*(*c*_*i*_, *c*_*j*_) is the Kronecker delta function, with *c*_*i*_ being the lattice to which the *i* node belongs to. The modularity compares the fraction of edges running between two vertices of the same type, with the fraction of such edges one would expect with a purely random distribution of edges among the entire network. In Table 4 we give the modularity values obtained in each experiment. In all cases, the *Q* value is positive, indicating a strong community structure inside the simulated network. This actually gives a clear indication about the modularity structure of the Italian population network, as encoded in the Polymod data, i.e. even if the Polymod survey does not furnish the structure of the contacts network, but only the number of contacts among different age classes, we see that in order to reproduce those contact patterns, we need to construct a network with a strong modularity character.

**Table 4:** Modularity.

**Simulations**	**D**	**Q**	**ΔQ**
**1 lattice**	6	0.648	0.010
**Experiment 1**	3	0.85487	9e-05
**Experiment 2**	4	0.85479	5e-05
**Experiment 3**	6	0.82748	6e-05
**Experiment 4**	8	0.82791	4 e-05

Modularity values in the 4 different simulated networks

### Epidemics Curves

In this section, we test the model against the Italian epidemiological data of the H1N1 pandemic during the season 2009/2010. We assume both the infective period, *T*_inf_, and the stop time, *T*_*s*_, to follow exponential distributions. As in [28], the average infective period is set equal to ${\bar{T}}_{inf}=5$ days, which corresponds to the typical duration of influenza symptoms (with a truncation of the distribution at $5{\bar{T}}_{inf}$), and the average stop time is set equal to ${\bar{T}}_{s}=1$ day, which corresponds to the typical duration of the asymptomatic phase for the influenza (with a truncation of the distribution at $5{\bar{T}}_{s}$). The simulations are initialized with a density of infected individuals, randomly distributed on the lattice, which is equal to the 5% of the density of infected individuals observed at 43-th week in the Italian epidemiological data of the H1N1 pandemic.

On each lattice, we set the parameters *S*_*a*_ (age-dependent susceptibility) in order to reproduce the peak of the outbreak in each age class. The values of the susceptibility, *S*_*a*_, obtained in the four lattice setup are roughly independent on *D*, and decrease with increasing age (*S*_1_ ∼ 0.07, *S*_2_ ∼ 0.04, *S*_3_ ∼ 0.025, *S*_4_ ∼ 0.012). This last result is in perfect agreement with the fact that the H1N1 virus caused symptomatic disease mainly in younger population, as a consequence of a pre-existing partial immunity of older people. In the single lattice topology [28], the susceptibilities do not have a monotonic behavior (*S*_1_ ∼ 0.07, *S*_2_ ∼ 0.12, *S*_3_ ∼ 0.019, *S*_4_ ∼ 0.004). In particular, in order to reproduce the peak of the outbreak in each age class, the susceptibility of the 5–14 years old people was chosen larger than the other ones. This choice allowed to compensate the fact that the number of daily contacts within the age class 5–14 was underestimated by the one lattice model. Here, instead, this effect is more realistically obtained combining the values of the mobilities with those of the susceptibilities. The 5–14 years old people is indeed the class with the largest value of the mobility (*mob*_1_ ∼ 0.64, *mob*_2_ ∼ 1, *mob*_3_ ∼ 0.23, *mob*_4_ ∼ 0.09), reflecting the fact that people at that age have the largest number of daily contacts.

**Fig 3:**
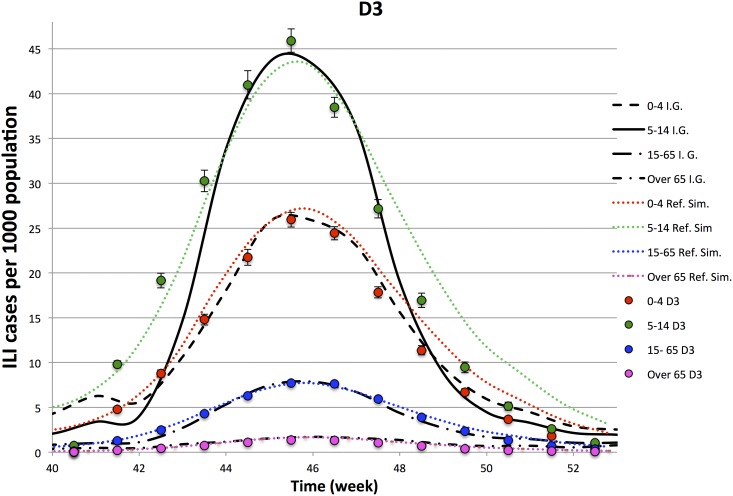
Epidemiological Curves—Simulation in D = 3.

**Fig 4:**
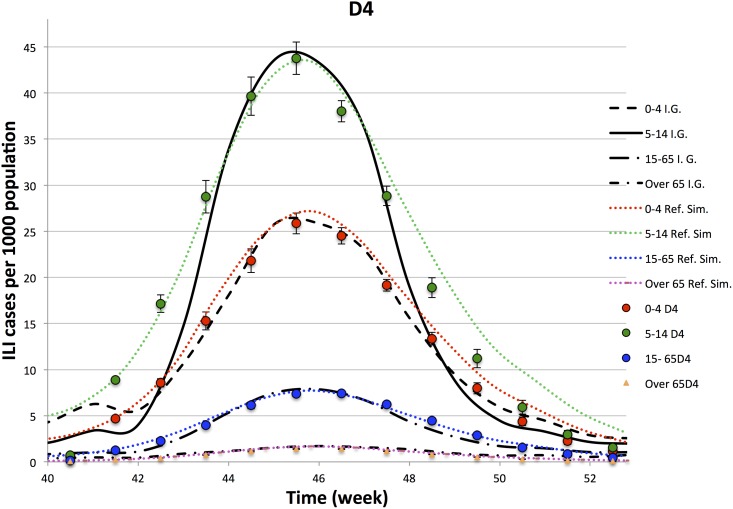
Epidemiological Curves—Simulation in D = 4.

**Fig 5:**
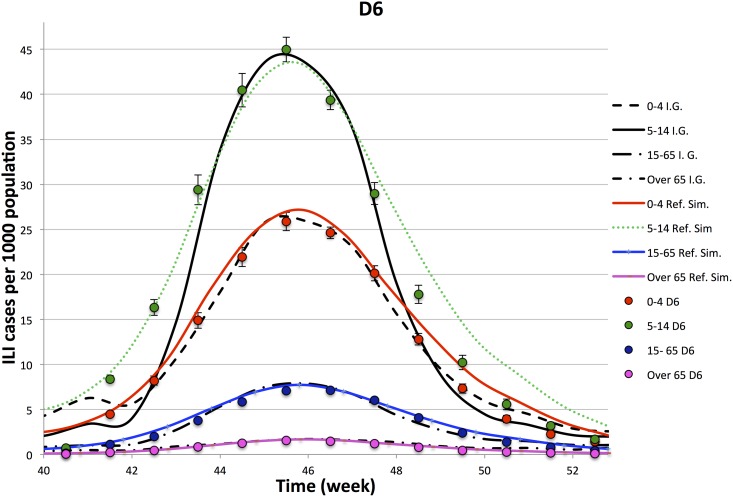
Epidemiological Curves—Simulation in D = 6.

**Fig 6:**
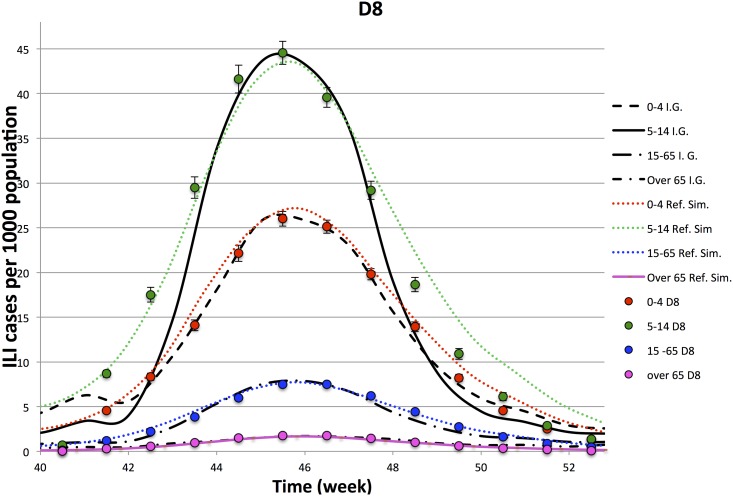
Epidemiological Curves—Simulation in D = 8.

In Figs 3–6, we compare the observed illness prevalence (per thousand of individuals) of H1N1 influenza cases in Italy from the 40-th week of the year 2009 to the simulated illness cases by age group, for *D* = 3, 4, 6, 8, respectively. In any cases, the agreement appears always better than in the single lattice setup. In particular the descendant phase of the epidemic curve for the class 5–14 significantly improves.

Figures show that the four lattice setup reproduces the epidemiological data for age classes 0–4, 15–64 and +65 in a long range of time (from week 41-th to week 51-th). However, it fails to reproduce the very beginning of the outbreak, and in minor measure the descendant phase, for the class 5–14. As already observed in Ref. [28], part of the disagreement at the beginning of the outbreak for the age class 5–14 (that is the one mostly involved into the epidemic) may be due to an underestimation of the influenza diffusion at that time, since not all the cases of influenza like symptoms led to illness consultations, as instead it mostly happened during the peak. Concerning the descendant phase, self-initiated health care measures carried out by many Italian families may explain the disagreement observed between simulated and observed cases in the class 5–14. Indeed, even if no closure of schools was decided by the Italian Government, the spread of fear during the peak (also due to a strong media campaign), induced many families to keep children at home, drastically reducing their scholar and extra scholar activities during that period.

In order to better analyze above findings, for each experiment, we perform a *χ*-square test on 9-points (from week 43.5 to week 51.5) and 11-points (from week 42.5 to week 52.5). Comparing different experiments, performed on the lattice dimensions, *D*, listed in Table 3, we see that

- Ordering the 4 experiments according to the goodness of the *χ*-square tests on the contact patterns, one also finds that they are in the same relation order of the *χ*-tests on the points of the epidemiological data.
- The 11-point *χ*-square tests of the epidemiological data have a higher variability with respect to the 9-point ones: i.e. the peak is quite well reproduced by all the experiments, while enlarging the size of the temporal window, including one week before and one week after the 9-point window, the *D* = 3 experiment gets worst.
- The comparison of the *χ*-tests of the four lattices topologies, with the single lattice topology, clearly shows a significant improvement generated by this new topology both on the epidemiological and contact patterns simulated data. Thus, an improvement in the accordance of the simulated contact patterns data with the Polymod data ensures an improvement also in the accordance of epidemiological data.

**Fig 7:**
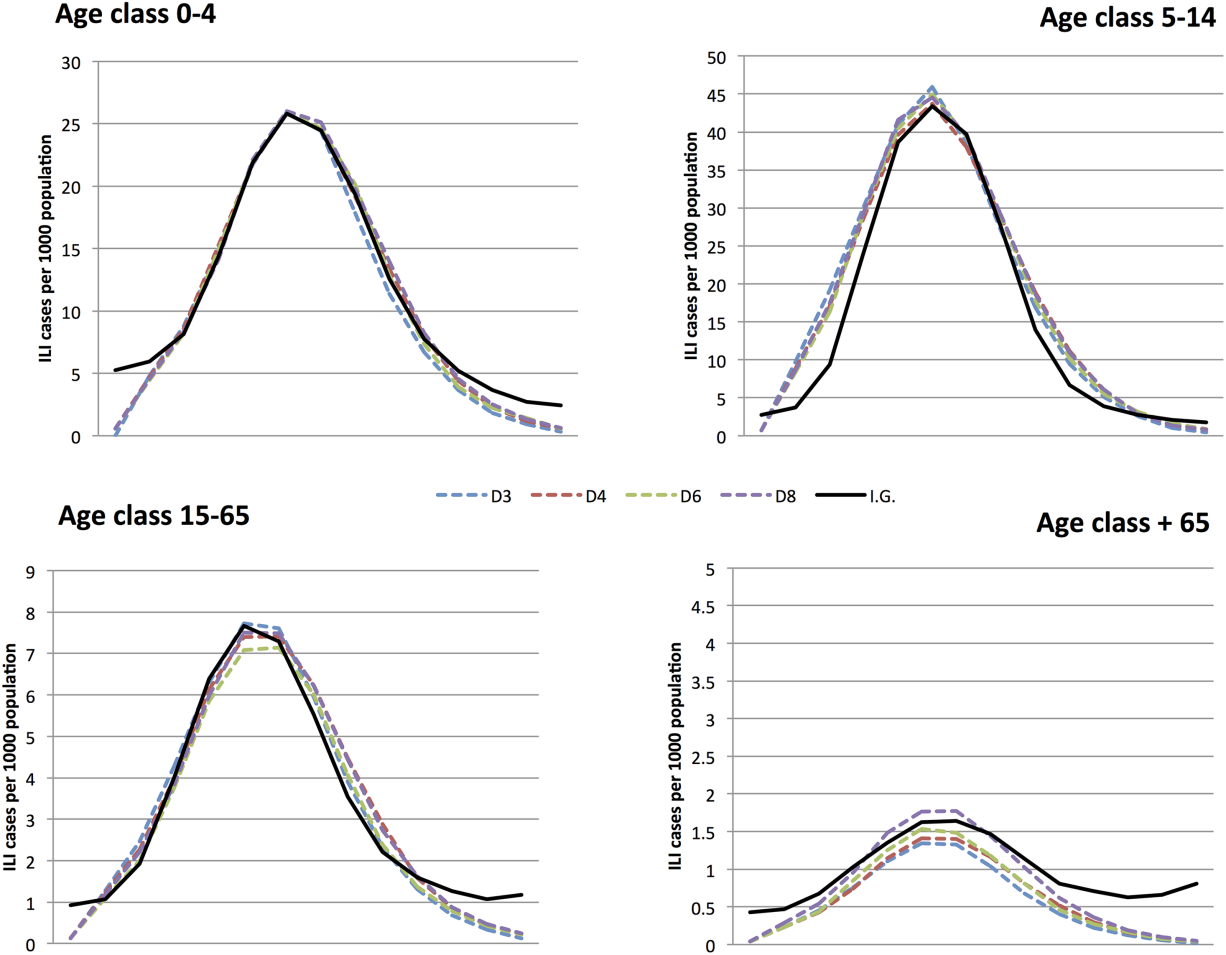
Age distribution of the ILI cases of the H1N1 pandemic in Italy during the season 2009/2010. Comparison between the epidemiological data, furnished by the Italian Government, and the simulated data in the four lattice setup with *D* = 3, 4, 6, 8.

Finally, in Fig 7, we compare the numerical data obtained in the four lattice setup for different lattice dimensions. In figure, the data on the H1N1 influenza cases in Italy are also shown. As we see, the data are qualitatively and also quantitatively similar, showing that the parameter *D* does not affect the influenza spreading at all. The observed differences may be interpreted as numerical artefact and could be further minimized by enhancing the statistics of the simulations. In this respect, it is worth noticing that the parameter *D* controls the topology of the space, in which particles move, and has no biological meaning: by changing its value, we change the topology of the contact network inside each age group. Essentially, different values of *D* correspond to different maximum numbers of simultaneous contacts that each individual may have in its age group (which is 2*D*, if all the nearest neighbor sites were occupied). This value does not influence directly the epidemic spreading, since we disregard the effect of the viral load in the infection process, which is indeed entirely ascribed to the immunological status of the susceptible individual. In particular, we do not consider the effect of the simultaneous presence of more than one infected individual in the neighborhood of the susceptible one. Thus, the probability that an individual is infected does not depend on the number of its nearest neighbors, which are simultaneously infected. Accordingly, in Table 3, one can notice that the differences in the *χ*-square tests for the contact patterns, obtained for different values of *D*, are very small. What is interesting, is that although small, differences on the *χ*-tests on contact patterns corresponds to differences between the *χ*-test on the epidemiological side.

## Discussion

In this paper, we propose a very simple model for the epidemic spreading in an age-structured population with dynamic contacts.

In particular, this work comes as a natural continuation of a series of papers, focused on a lattice gas model for the epidemic spreading [27–29]. The novelty of the present model is the lattice topology, which indeed is a four-lattices topology with individuals of different age classes living on different lattices. The four lattices setup has many advantages: first of all, it introduces a community structure in the contact network and this allows to fix separately the internal and external contacts, introducing different parameters to regulate them. This circumstance is crucial in order to reproduce the full contact matrix and not only the overall number of contacts of individuals of a certain age, which was indeed a limit of the previous approach.

Furthermore, from the statistical mechanics point of view, the four lattice system is definitely simpler than the single lattice model with four different species living on the same lattice. Indeed, as discussed in Ref. [28], in the single lattice model the system can be viewed as a mixture of 4 species of particles, with an attractive interaction between particles of the same type, regulated by a set of parameters *β*_age_. In the presence of a single specie, at low *β* (corresponding to high temperatures), the system is in a homogeneous disordered phase. After a quench at high *β*_age_ (low temperatures), domains of particles of the same type appear. As time passes, the typical size of the domains slowly increases. The presence of different species, that interact by means of the excluded volume, further complicates the dynamics, since isolated particles in domains of a different type may remain blocked in this state for a long time, although this is not an equilibrium state for the system. In particular, different quenching procedures, in general, produce different final states, for the same set of dynamical parameters, *β*_age_, and therefore the final state, although stationary on our observation time scale, may be an out-of equilibrium state.

In the new setup, instead one has four distinct lattices each hosting one specie of particle moving on it at random. In this case, the thermalization procedure is extremely simple since each age class reaches the equilibrium state separately and particles on each lattice interact only with excluded volume. With this topology, the risk that the system is blocked in a non equilibrium state for a long time is completely avoided. As a consequence, the model is stable with respect to the thermalization procedure, as well as to modifications of different parameters, such as the lattice dimension.

The comparison among experiments with different values of *D* shows clearly that the lattice dimensions, which allow to better reproduce the contact matrix, are also those that correspondingly give the best results for epidemic spreading. This is an explicit confirmation of the social contact hypothesis: a better accordance of the contact patterns of the model with gathered data ensures a better accordance also on the epidemiological side. This result shows how rich is the information encoded in the age-structured average contact patterns of individuals with respect to the analysis of the epidemic spreading of an infectious disease.

Finally, in the present model the epidemic spreading takes place on a heterogeneous contact network, in which individuals have a variable number of contacts with variable identities. The literature on epidemic models formulated on complex networks (e.g. [38–45]) shows the importance of the network structure in determining the epidemic dynamics [46–48]. Correlations among the node connectivity and among node status on the epidemic dynamics has also been investigated [49–53]. As a future possible development, it would be interesting to apply those correlation approaches to our model.
